# Editorial: The sociology of gambling

**DOI:** 10.3389/fsoc.2024.1387929

**Published:** 2024-03-13

**Authors:** Michael Egerer, Søren Kristiansen, Virve Marionneau, Jani Selin, Johanna Järvinen-Tassopoulos

**Affiliations:** ^1^Faculty of Social Sciences, University of Helsinki, Helsinki, Finland; ^2^Department of Sociology and Social Work, Aalborg University, Aalborg, Denmark; ^3^University of Helsinki, Helsinki, Finland; ^4^National Institute for Health and Welfare, Helsinki, Finland

**Keywords:** gambling, sociology, gambling (gaming), gambling attitudes, social norms, theory

Gambling has been a prominent part of many classic sociological texts, from Simmel (1983 [1922]) essay on the philosophy of adventure and Veblen's (1899) analysis of the leisure class, to Huizinga (2016 [1938]) “Homo Ludens,” Caillois's (1958) sociology of play, and Goffman's (2006 [1967]) essay “Where the Action Is.” More recently, several contemporary sociologically oriented gambling scholars have further developed the sociological understanding of gambling by applying existing social theory, and developing gambling-specific theoretical approaches. Today, the sociology of gambling is an ever-growing field in which sociological theory is applied to understanding gambling behavior, industry practices, and regulatory strategies. This insight has challenged traditional psychological approaches to the field and expanded our sociological understanding of consumption and addiction. The Frontiers Research Topic on “*The sociology of gambling*” outlines a collection of sociological research on gambling, with the aim of integrating the sociology of gambling into the wider field of sociological theory.

Gambling in its multifarious dimensions plays on all registers of the sociological score. It taps into sociological constructs such as power, inequality, risk, social structure, and social interaction. It also invokes Mills' (1959) sociological imagination, with its suggestion of linking social issues to personal troubles. Gambling spans a network of relations, be they interpersonal, institutional, or related to objects. These networks and relationships are also visible in the current collection.

In their study of sportswashing, Syvertsen et al. demonstrate the interrelations between gambling and sport for gamblers, industry actors, and political actors. The article argues that betting cannot be separated from sports spectatorship. Instead, these two are interrelated. Sport is a prerequisite for sports betting, but it also serves social or even political functions. While the ‘societal context' often serves as a catch-all concept for external risk factors in non-sociological research, the sociological perspective can incorporate the setting as inherently interwoven with gambling and its risks.

The context of gambling is also highlighted in the work on problem gambling in Tonga by Fehoko et al.. Rather than considering culture and gender as risk factors, as is the tradition within the Western concept of individual rationality, this contribution takes a phenomenological approach using the *talanoa* research method. This approach allows for an understanding of gambling and the risks related to it, in relation to the obligations of young men in the Tongan community and the achievement of status within the community's family networks.

Drawing on a European sociological tradition, the importance of the family as a system is also at the core of Egerer's contribution to this collection. Egerer uses Niklas Luhmann's systems theory to establish a typology of gambling as behavior, breaking away from previous actor-centered typologies of gamblers.

Selin focuses on the relationship between gamblers and corporations by discussing the responsible gambling practices of gambling companies as a production of subjectivities in gamblers. Gambling has always been an object of social control. Using Michel Foucault's approach to governmentality, Selin shows how gambling companies share a hesitancy to intervene in gamblers' behavior, as subjective self-control is deeply embedded in their “responsible gambling” practices.

“Responsible gambling” is also the focus of Marko et al.' study of people with lived experience of gambling harm. While the “responsible gambling” paradigm is constructed to assume that gamblers are rational consumers, this study shows how this focus on individual self-control is also connected to unintended side effects, including stigmatization or even self-stigmatization. The authors call for more attention to messaging strategies to avoid blaming those who engage in gambling for harm, and to advocate for a more comprehensive public health approach that also considers gambling environments.

The relationship between gamblers and the industry is discussed by Pitt et al. in their study of the gambling environment of older Australian gamblers. Based on their results, the decision to engage in gambling is not a natural drive but emerges in the context of technological developments, industry strategies and marketing, and regulatory choices.

Gambling itself can also have different meanings. Gambling can constitute a leisure activity for some, but it can also be considered “work” for others. Weidner's study looks at the financial market, with its speculative nature of trading as an expression of the essence of gambling. Comparing the regulatory approaches of the two, Weidner shows that while the guiding principle of gambling industry regulation is player protection, the regulation of the financial market aims at informing market participants.

Finally, gambling is at the forefront of the development of the digitalization of social life. The digital economy transverses borders, and escapes the control of nation-bound regulation. Järvinen-Tassopoulos et al. look at the concept of channelization—a regulatory principle aimed at curbing offshore markets for online gambling. Using interview data with representatives of the Finnish gambling monopoly and regulators, the authors demonstrate how channelization has become a policy aim rather than a tool. This risks obscuring the consumer protection issues that initially motivated channelization policies.

This Research Topic provides an example of the ways in which sociology can inform gambling research. It exemplifies how gambling is deeply rooted in human society. At the same time, this collection is only the beginning of future sociological research on gambling and gambling research in sociology.

## Author contributions

ME: Writing – original draft, Writing – review & editing. SK: Writing – original draft, Writing – review & editing. VM: Writing – original draft, Writing – review & editing. JS: Writing – original draft, Writing – review & editing. JJ-T: Writing – original draft, Writing – review & editing.
